# Adjuvanted *Schistosoma mansoni*-Cathepsin B With Sulfated Lactosyl Archaeol Archaeosomes or AddaVax™ Provides Protection in a Pre-Clinical Schistosomiasis Model

**DOI:** 10.3389/fimmu.2020.605288

**Published:** 2020-11-16

**Authors:** Dilhan J. Perera, Adam S. Hassan, Yimei Jia, Alessandra Ricciardi, Michael J. McCluskie, Risini D. Weeratna, Momar Ndao

**Affiliations:** ^1^ Division of Experimental Medicine, Department of Medicine, McGill University, Montreal, QC, Canada; ^2^ Infectious Diseases and Immunity in Global Health Program, The Research Institute of the McGill University Health Centre, Montreal, QC, Canada; ^3^ Department of Microbiology and Immunology, McGill University, Montreal, QC, Canada; ^4^ Human Health Therapeutics Research Centre, National Research Council Canada, Ottawa, ON, Canada; ^5^ National Reference Center for Parasitology, Research Institute of the McGill University Health Centre, Montreal, QC, Canada

**Keywords:** *Schistosoma mansoni*, schistosomiasis, vaccine, adjuvant, archaeosomes, AddaVax

## Abstract

Schistosomiasis threatens 800 million people worldwide. Chronic pathology manifests as hepatosplenomegaly, and intestinal schistosomiasis caused by *Schistosoma mansoni* can lead to liver fibrosis, cirrhosis, and blood in the stool. To assist the only FDA-approved drug, praziquantel, in parasite elimination, the development of a vaccine would be of high value. *S. mansoni* Cathepsin B (SmCB) is a well-documented vaccine target for intestinal schistosomiasis. Herein, we test the increased efficacy and immunogenicity of SmCB when combined with sulfated lactosyl archaeol (SLA) archaeosomes or AddaVax™ (a squalene based oil-in-water emulsion). Both vaccine formulations resulted in robust humoral and cell mediated immune responses. Impressively, both formulations were able to reduce parasite burden greater than 40% (WHO standard), with AddaVax™ reaching 86.8%. Additionally, SmCB with both adjuvants were able to reduce granuloma size and the amount of larval parasite hatched from feces, which would reduce transmission. Our data support SmCB as a target for *S. mansoni* vaccination; especially when used in an adjuvanted formulation.

## Introduction

Schistosomiasis (Bilharzia) is an underestimated parasitic disease for which over 800 million people are at risk (1). This blood fluke spreads through fresh water in tropical and sub-tropical regions. Adult worms cause little to no pathology (2), however, female worms lay hundreds to thousands of eggs per day depending on the species of *Schistosoma*, some of which exit with the feces or urine, and others become trapped in host tissues causing chronic pathology.

Praziquantel (PZQ) used for the treatment of schistosomiasis has a reported efficacy of 86–93% (3, 4). However, it does not protect individuals from reinfection or remove pre-existing egg deposition. To aid the interruption of schistosomiasis a vaccine is pertinent (5). In the 1990s, independent testing of six candidate *S. mansoni* antigens underwent protective studies organized by a UNDP/World Bank/WHO Special Programme for Research and Training in Tropical Diseases (TDR/WHO) committee. Although these trials resulted in protection, the WHO goal of 40% or greater protection was not met, headlining the need for possible adjuvanted formulations (6).

*S. mansoni* Cathepsin B (SmCB) is the most abundant cysteine protease found in schistosomula and adult worm gut and somatic extracts. This protein is used for host blood molecule degradation and nutrient acquisition (7, 8). RNA interference studies demonstrate that when cathepsin B transcript levels are suppressed resulting worms show significant growth retardation compared to control parasites (9). By targeting cathepsin B, reduced egg fitness has been demonstrated by our group (10), and parasite anti-fecundity has also been seen in other flukes (11).

Our lab has exploited the immunogenic gut peptidase SmCB as a vaccine target, which reduces worm parasite burden by 59 and 60% when adjuvanted with CpG dinucleotides, and Montanide ISA 720 VG, respectively (12, 13).We believe that by combining this antigen with novel adjuvants, we will be able to increase parasite burden reduction and develop a more promising anti-schistosomiasis vaccine. Herein, we evaluate the immunogenicity and protective capability of our recombinant SmCB (rSmCB) adjuvanted by two additional adjuvants namely sulfated lactosyl archaeol (SLA) archaeosomes, and AddaVax™ (AddaVax).

## Methods

### SmCB Recombinant Protein Preparation

*S. mansoni* Cathepsin B was prepared and purified as we previously described (12). Briefly, the PichiaPink™ system (Thermo Fisher Scientific, Waltham, MA, USA) was used, and recombinant yeast cells were cultured in a glycerol medium. After 3 days of growth, yeast cells were resuspended in a methanol induction medium to allow expression of recombinant protein. Recombinant protein purification was performed by Ni-NTA chromatography (Ni-NTA Superflow by QIAGEN, Venlo, Limburg, Netherlands). The eluted protein was analyzed by Western Blot using antibodies directed at the His-tag.

### Immunization Protocol

Six to eight-week old female C57BL/6 mice were bred from mice obtained from Charles River Laboratories (Senneville, QC). Four groups of mice (n = 10–13) were immunized for humoral and cytokine assessment. Four groups of mice, (n = 10) were immunized and subsequently infected for parasite burden assessment. Group 1 (control): Mice were injected with phosphate-buffered saline (PBS) (Wisent Bioproducts, St. Bruno, QC). Group 2 (positive control): Mice were immunized with 20 μg of recombinant SmCB (rSmCB) and 35μl of Montanide ISA 720 VG (SEPPIC Inc., Fairfield, NJ). Group 3: Mice were immunized with 20 μg of rSmCB admixed with 1 mg of pre-formed empty SLA archaeosomes (NRC, Ottawa, Canada). Group 4: Mice were immunized with 20 μg of rSmCB and 25 μl of AddaVax™ (InvivoGen, San Diego, CA). Each mouse was immunized at weeks 0, 3, and 6 intramuscularly in the thigh with 50 μl of vaccine.

### *Schistosoma mansoni* Challenge

*Biomphalaria glabrata* snails infected with the Puerto Rican strain of *S. mansoni* were provided by NIAID Schistosomiasis Resource Center of the Biomedical Research Institute (Rockville, MD). Three weeks after the final immunization mice were challenged with 150 cercaria *via* tail exposure for 1 h and sacrificed 7 weeks later for parasitological measures. Images of mouse livers were taken during dissection using a Galaxy S8 cell phone camera (Samsung Group, Seoul, South Korea). Adult worms were perfused from the hepatic portal system and counted manually (13). Liver sections were suspended in 10% buffered formalin phosphate (Fisher Scientific) and processed for histology as previously described (10). Remaining liver and intestines were weighed and digested overnight in 4% potassium hydroxide. The following day, the eggs present in these tissues were counted by microscopy and adjusted per gram of tissue. Burden reductions were calculated as previously described (12–14):

$$\text{Percent}\;\text{of}\;\text{worms}\;\text{or}\;\text{eggs}\;\text{reduction}=\left(1-\frac{\text{mean}\;\text{number}\;\text{of}\;\text{worms}\;\text{or}\;\text{eggs}\;\text{recovered}\;\text{in}\;\text{immunized}\;\text{mice}}{\text{mean}\;\text{number}\;\text{of}\;\text{worms}\;\text{or}\;\text{eggs}\;\text{recovered}\;\text{in}\;\text{PBS}\;\text{control}\;\text{mice}}\right)\times 100$$

### Serum Total SmCB-Specific IgG

SmCB-specific serum IgG was assessed by ELISA as described elsewhere (10). Briefly, high binding 96-well plates (Greiner Bio-One, Frickenhausen Germany) were coated with rSmCB (0.5 μg/ml) in 100 mM bicarbonate/carbonate buffer (pH 9.6) overnight at 4°C. After blocking plates with 2% bovine serum albumin (BSA; Sigma Aldrich) in PBS-Tween 20 (PBS-T: 0.05%; Fisher Scientific, Ottawa, ON, Canada) (blocking buffer), serum samples were added to the plates in duplicate. Plates were incubated for 1 h at 37°C then washed with PBS (pH 7.4) and horseradish peroxidase (HRP)-conjugated anti-mouse IgG (Sigma Aldrich) was diluted 1:20,000 in blocking buffer and applied. Again, plates were washed with PBS, and 3,3’,5,5’-tetramethyl benzidine (TMB) substrate (Millipore, Billerica, MA) was used for detection followed by the addition of H_2_SO_4_ (0.5M; Fisher Scientific). Optical density (OD) was measured at 450 nm with an EL800 microplate reader (BioTek Instruments Inc., Winooski, VT), and concentration of SmCB specific IgG was calculated by extrapolation from the IgG standard curve.

### Serum SmCB-specific IgG1 and IgG2c

SmCB-specific serum IgG1, and IgG2c were assessed by ELISA as described elsewhere (10). Briefly, Immulon 2HB flat-bottom 96-well plates (Thermo Fisher) were coated with recombinant SmCB (0.5 μg/ml) in 100 mM bicarbonate/carbonate buffer (pH 9.6). The plates were washed with PBS-T, and blocking buffer was applied for 90 min. A serial dilution of serum was applied to plates in duplicate and incubated for 2 h at 37°C. Plates were washed again with PBS-T, and goat anti-mouse IgG1-HRP (Southern Biotechnologies Associates, Birmingham, AL) or goat anti-mouse IgG2c-HRP (Southern Biotechnologies Associates) was applied to plates for 1 h at 37°C. After a final wash, TMB was added followed by H_2_SO_4._ Again, OD was measured as above. IgG1 and IgG2c endpoint titers were calculated as the reciprocal of the highest dilution, which gave a reading above the cut-off. The endpoint titer cut-off was statistically established as described elsewhere (15) using the sera of PBS immunized, unchallenged mice.

### Serum Total IgE

Total IgE was assessed by ELISA using the BD OptEIA™ Set Mouse IgE Kit (BD, San Diego, CA) following manufacturer’s guidelines. Briefly, high binding 96-well plates (Grenier Bio-One) were coated with anti-mouse IgE capture antibody diluted in 100 mM bicarbonate/carbonate buffer (pH 9.6) 250-fold. Plates were again washed. Plates were washed and then blocked using PBS with 10% fetal bovine serum (Wisent Bio Products) for 1 h at room temperature. Plates were again washed. Samples were diluted in assay diluent then added to plates with standards and incubated for 2 h at room temperature. Plates were washed again, and biotinylated anti-mouse IgE antibody and streptavidin-horseradish peroxidase were added together for 1 h at room temperature. Plates were then washed a final time before TMB was added for 30 min protected from light. Lastly, 50 μl of H_2_SO_4_ was added to wells, and absorbance was read at 450 nm within 30 min.

### Cell-Mediated Immune Responses

Three weeks after the last immunization, mice were sacrificed, spleens collected, and splenocytes isolated as previously described (16) with the following exceptions: splenocytes were resuspended in RPMI-1640 supplemented with 10% fetal bovine serum, 1 mM penicillin/streptomycin, 10 mM HEPES, 1X MEM non-essential amino acids, 1 mM sodium pyruvate, 1 mM L-glutamine (Wisent Bioproducts), and 0.05 mM 2-mercaptoethanol (Sigma Aldrich) (fancy RPMI, fRPMI). These cells were then used in the following assays:

### Proliferation Assay by BrdU

Cell proliferation was measured by using the Roche chemiluminescent kit, following manufacturer’s guidelines. Splenocytes were seeded in black 96-well flat bottom plates at 200 000 cells per well. Each sample was seeded unstimulated, stimulated with rSmCB (2.5 μg/ml), and stimulated with concavalin A (2.5 μg/ml) as a positive control. Briefly, cells were incubated for 48 h at 37°C and 5% CO_2_. At this time, 20 μl of BrdU labeling reagent was added to each well after being diluted 1:100 in fRPMI, and cells were incubated again for another 24 h. Cells were resuspended in 200 μl of PBS as a wash step, and then dried for 1 h in a 60°C hybridization oven (Thermo Fisher). Carefully 200 μl of FixDenat was added to each well for 30 min at room temperature, before 100 μl of Anti-BrdU-POD working solution (1:100 in antibody dilution solution) was added for an additional 90 min. Plates were washed three times with washing solution, and 100 μl/well of substrate solution was added. The plate was protected from light and shaken for three min before light emission was measured using a Tecan Infinite^®^ 200 PRO (Tecan, Switzerland) within 10 min.

### Cytokine Production by Multiplex ELISA

Splenocytes were incubated at 300 000 cells in 200 μl with rSmCB in fRPMI (2.5 μg/ml recombinant protein). After 72 h at 37°C + 5% CO_2,_ plates were centrifuged and supernatant collected and stored at -80°C until analysis. Cell supernatants were assessed for the presence of 16 cytokines and chemokines [IL1-a, IL1-b, IL-2, IL-3, IL-4, IL-5, IL-6, IL-10, IL-12p70, IL-17, IFNy, TNFa, CCL2 (MCP-1), CCL3 (MIP-1a), CSF2 (GM-CSF), and CCL5 (RANTES)] using Q-plex Mouse Cytokine–Screen (16-plex) multiplex ELISA following the manufacturer’s guidelines (Quansys Biosciences, Logan, UT, USA). Samples were run in singlet.

### T-Cell-Mediated Cytokine Secretion by Flow Cytometry

Splenocytes were seeded into 96-well U-bottom plates (BD Falcon) at 10^6^ cells in 200 µl/well. Duplicate cultures were stimulated with or without rSmCB in fRPMI (2.5 μg/ml) for 24 h at 37°C + 5% CO_2_. For the last 6 h of incubation, protein transport inhibitor was prepared according to the manufacturer’s guidelines (BD Science, San Jose, CA) and added to all samples. Cells stimulated with phorbol 12-myristate 13-acetate and ionomycin were processed as positive controls. Plates were then processed for flow cytometry as described elsewhere (17). Briefly, splenocytes were washed twice with 200 μl of cold PBS, and fixable viability dye eFluor 780 (Affymetrix ebioscience, Waltham, MA) was applied at 50 μl/well diluted at 1:300 and incubated for 20 min at 4°C protected from light. Cells were washed as above with PBS 1% BSA (PBS-BSA), and Fc block (BD Science) diluted 1:50 was added for 15 min. All surface stains were diluted 1:50 in PBS-BSA and 50 μl/well of extracellular cocktail was applied for 30 min at 4°C protected from light. The following antibodies made up the extracellular cocktail: CD3-FITC (Clone 145-2C11, Affymetrix ebioscience), CD4-V500 (RM4-5, BD Bioscience), and CD8-PerCP-Cy5 (Clone:53-6.7, BD Science). Cells were then washed as above with 1X fixation buffer (BD Science) and left overnight at 4°C in the dark. Plates were washed as before with 1X permeabilization buffer (BD Science) and stained with an intracellular cocktail of antibodies diluted 1:50 in PBS-BSA applied as 50 μl/well for 30 min at 4°C protected from light. The intracellular cocktail was made up of: IL-2-Pe-Cy5 (Clone: JES6;5H4, Biolegend, San Diego, CA), IFNγ-PE (Clone: XMG1.2, BD Science), and TNFα-efluor450 (Clone: MP6-XT22, Affymetrix ebioscience). After staining, cells were resuspended in PBS and analyzed on BD LSRFortessa X-20 (BD Science) using Flowjo software (version 10.0.8r1). Our gating strategy is shown in **Supplemental Figure 1**.

### Histology and Egg Granuloma Quantitation

Liver sections in 10% buffered formalin phosphate were stained using hematoxylin and eosin to assess granuloma size and egg morphology. Granuloma area was measured using Zen Blue software (version 2.5.75.0; Zeiss) as previously reported (10, 18–21). Briefly, while working at 400x magnification, the pointer was used to trace the perimeter of 37–41 granulomas per experimental group with a clearly visible egg which the software converted into an area. Hepatic eggs were classified as abnormal if their internal structure was lost or the perimeter of the egg was crenelated. Fifteen different fields of vision were assessed per experimental group over two independent experiments. Abnormal eggs were counted and reported as a percent of the total eggs counted per field of vision.

### Miracidia Hatching Experiments

Miracidia hatching was optimized and adapted from a protocol as described elsewhere (22).

Briefly, one gram of feces from each experimental group was collected twice, one day before and at sacrifice 7 weeks post infection. Feces from each time point were assessed individually. Feces were resuspended in distilled water and transferred into an Erlenmeyer flask/conical tube. The flask/tube was then wrapped in tin foil to protect from light and was topped up with distilled water so that only 3 mm under the lid was exposed to light. Tin foil wrapped flasks were placed inside of a cardboard box, with a hole the same diameter as a lamp, through which light was shone on them for 3 h. After this time, water samples were collected from the exposed fraction of water and miracidia were counted. An image of the set up, and further detailed methodology can be seen in **Supplemental Figure 2**.

### Statistical Analysis

Statistical analysis was performed using GraphPad Prism 6 software (La Jolla, CA). Data were analyzed by Kruskal Wallis one-way ANOVA with Dunn’s multiple comparisons tests. Flow cytometry data were analyzed by a two-way ANOVA and Dunnett’s multiple comparisons tests. If present, outliers were calculated using GraphPad QuickCalcs and removed. P values < 0.05 were considered significant.

## Results

### Humoral Response to Vaccination

**Figure 1:**
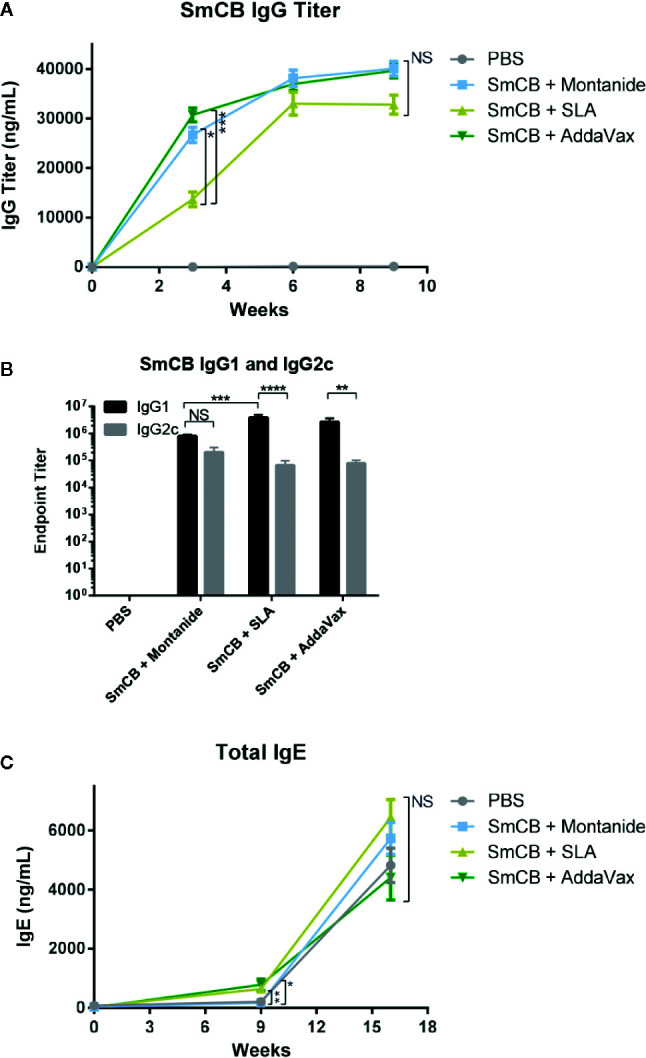
Humoral response. Production of SmCB specific total IgG n = 20 from four independent experiments **(A)**, Antigen specific IgG1 and IgG2c n = 10 from two independent experiments **(B)** in immunized mice. Production of total IgE n = 10 from two independent experiments **(C)** in immunized and challenged mice. Graphs A and C show antibody titers for PBS control mice, rSmCB and Montanide, SLA, and AddaVax. Graph B shows the endpoint titer of SmCB specific IgG1 and IgG2c at week 9, in black and gray, respectively. Serum from individual mice was analyzed by ELISA. Means and SEM are shown. NS, not significant, *P < 0.05, **P < 0.01 ***P < 0.001, ****P < 0.0001.

No mice had detectable SmCB-specific IgG antibodies at baseline, and the PBS control remained negative throughout the study (**Figure 1A**). Mice receiving adjuvanted rSmCB developed SmCB-specific IgG after a single immunization. At week 3, groups adjuvanted with Montanide and AddaVax had significantly higher titers than with SLA, however this difference was no longer significant post first boost. Antigen specific IgG titers in vaccinated mice rose until week 6 before plateauing.

Endpoint titers were calculated for antigen specific IgG1 and IgG2c at the time of infection (**Figure 1B**). Each experimental group elicited a robust mixed IgG1/IgG2c response, although mice vaccinated with antigen and SLA or AddaVax had much higher IgG1 (3.84e6 ± 1.13e6 and 2.69e6 ± 9.24e5, respectively, than IgG2c titers (6.60e4 ± 3.21e4 and 7.88e4 ± 2.38e4, respectively). Mice immunized with rSmCB/Montanide had a balanced IgG1/IgG2c response with titers of 8.00e5 ± 1.09e5 and 2.03e5 ± 9.75e4, respectively. At this time, and at the second study endpoint (post-challenge) mouse serum was also analyzed for total IgE (**Figure 1C**). At baseline, mice had little to no detectable IgE. In comparison to PBS controls, there was a greater increase in total IgE levels post immunization in groups receiving rSmCB adjuvanted with SLA (~3 fold) or AddaVax (~3.5 fold) than in the group immunized with rSmCB adjuvated with Montanide which saw no increase. Upon parasite challenge, the total IgE titers increased in all groups including the PBS controls with no significant differences between groups.

### Lymphoproliferation, Splenocyte Cytokine, and Chemokine Production in Response to Vaccination

**Figure 2:**
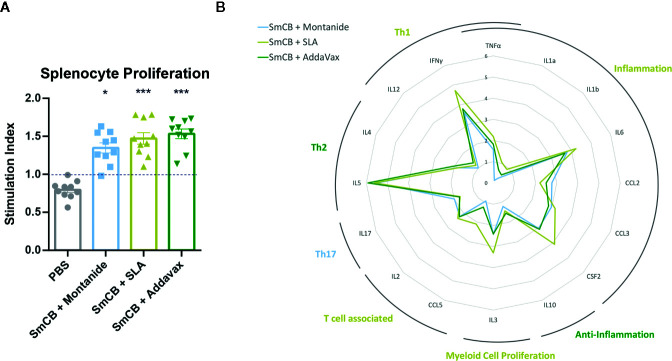
Lymphoproliferation, cytokines, and chemokines. Splenocyte proliferation shown as stimulation index in response to rSmCB restimulation *ex vivo* **(A)**. Means are shown with SEM. Significance is calculated against the PBS control. Mean levels of cytokine and chemokine expression were also reported as the fold change above the PBS control group and depicted in the radar plot in **(B)** with the axis in the natural log. Cytokines and chemokines have been grouped according to general functionality and labelled accordingly. Labels are colored reflecting the experimental group expressing the most amount of their cytokines/chemokines. n = 10 from two independent experiments. *P < 0.05, ***P < 0.001.

Enhanced SmCB-specific lymphoproliferation was seen in in *ex vivo* stimulated splenocytes from immunized compared to control mice. However, no statistical differences in the magnitude of lympho-proliferation was observed between immunized groups (**Figure 2A**). Differences in functionality of antigen-specific lymphocytes were further assessed by measuring cytokine and chemokine concentrations in culture supernatants.

For many of the cytokines and chemokines tested, adjuvanted formulations generated elevated levels above the PBS control (**Supplemental Figure 3**). However, differences can be seen in the cytokine milieus between experimental groups. The fold change expression of each cytokine/chemokine from the PBS control is depicted in a radar plot (**Figure 2B**) indicating each vaccine formulation favors a slightly different immune phenotype. Montanide has an increased Th17 immune profile, SLA an inflammatory, Th1, T-cell associated, and myeloid proliferating profile, and AddaVax a Th2 and anti-inflammatory profile.

### T-Cell Th1 Response to Vaccination

**Figure 3:**
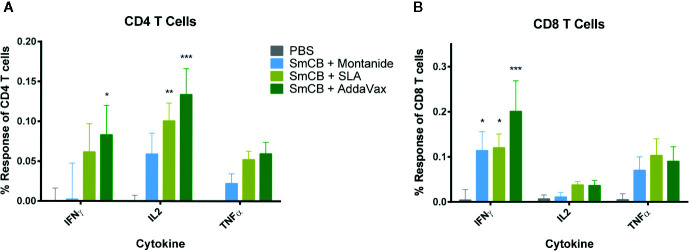
CD4+ and CD8+ T cell response. Splenocytes were restimulated with rSmCB *ex vivo* and CD4+ **(A)** and CD8+ T cells **(B)** were assessed for their expression of IFNγ, IL-2, and TNF-α. Means and SEM of subtractive data are shown (stimulated cells – unstimulated cells). The PBS control group is shown in gray, Montanide, SLA, and AddaVax groups are shown in blue, light green, and dark green, respectively. Significance is calculated against the PBS control. n = 13 from three independent experiments. *P < 0.05, **P < 0.01, ***P < 0.001.

Flow cytometry was used to enumerate splenic CD4+ and CD8+ T cell expression of IFNγ, IL-2, and TNFα in response to SmCB. Overall, an increase in cytokine expression was observed in CD4+ (**Figure 3A**) and CD8+ (**Figure 3B**) T cells in groups immunized with adjuvanted rSmCB over PBS controls. Mice immunized with rSmCB adjuvanted with SLA showed a significant increase in CD4+ IL-2 expression, whereas mice immunized with rSmCB adjuvanted with AddaVax showed a significant increase in CD4+ IL-2 and IFNγ expression compared to PBS control mice. All groups receiving adjuvanted rSmCB showed a significant increase in CD8+ IFNγ expression.

### Protection From Infection Upon Immunization With Adjuvanted rSmCB

**Figure 4:**
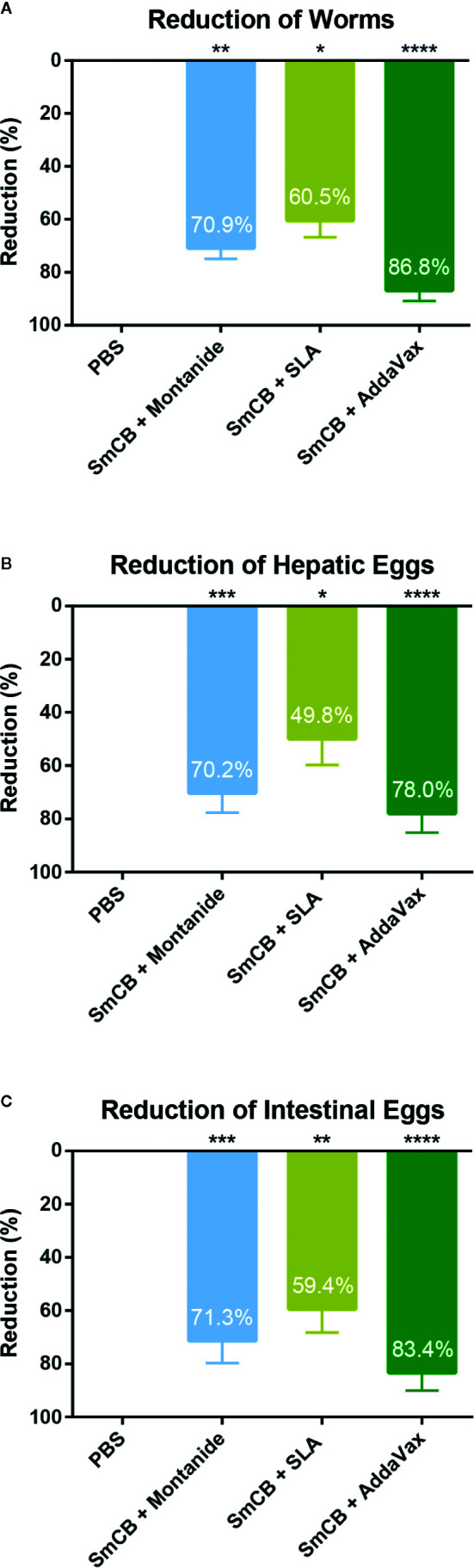
Parasitological outcomes. Seven weeks after challenge, mice were euthanized, and worms and eggs were counted for parasite burden. Parasite burden reductions are shown as mean and SEM for adult worms **(A)**, hepatic eggs **(B)**, and intestinal eggs **(C)**, eggs adjusted per gram adjusted per gram of tissue. Significance is calculated against the PBS control. n = 10 from two independent experiments. *P < 0.05, **P < 0.01, ***P < 0.001, ****P < 0.0001.

To determine the protective potential of the vaccines, a three-dose immunization regiment was tested. The average amount of worms collected from PBS control mice was 31 ± 7 worms over two independent experiments. Parasite burden reductions were calculated in reference to the PBS control mice within the same experiment to keep consistency within batches of infections. Parasite burden reductions were then combined and compared. All vaccine formulations significantly reduced parasite burden over PBS control with percent reduction in worm burden of 70.9 ± 3.9%, 60.5 ± 6.3%, and 86.8 ± 4.0% in groups adjuvanted with Montanide, SLA and AddaVax, respectively (**Figure 4A**). There were no statistical differences in burdens between the three formulations.

Pathology in schistosomiasis is caused by parasite eggs which become trapped in host tissues. Egg burdens in the liver (**Figure 4B**) and intestines (**Figure 4C**) were also calculated. Hepatic eggs in the PBS control group varied between 1250 and 14525 eggs/gram liver tissue. Similarly, intestinal eggs ranged between 1660 and 16973 eggs/gram intestine. rSmCB/Montanide reduced parasite burden by 70.3 ± 7.4% and 71.3 ± 8.4% in hepatic and intestinal eggs, respectively. The formulation of rSmCB/SLA reduced parasite burden by 49.8 ± 9.9% and 59.4 ± 8.8%, while rSmCB/AddaVax reduced parasite burden the most significantly, 78.0 ± 7.2% and 83.4 ± 6.6%, in hepatic and intestinal eggs, respectively.

### Liver Pathology

**Figure 5:**
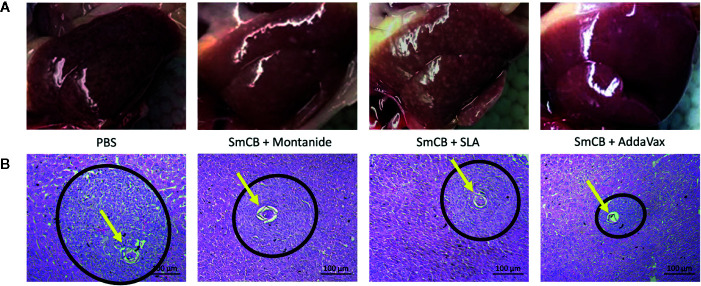
Liver pathology. Images of gross livers were taken **(A)**, and liver sections were stained by H&E **(B)**. In A, representative liver images from two independent experiments are shown for the PBS control on the left, and the experimental groups from left to right: Montanide, SLA, and AddaVax. Below, in B, H&E staining of hepatic tissue shows an *S. mansoni* egg (pointed to with a yellow arrow) within a granulomatous formation (within a black circle). H&E stained slides were viewed at 400X.

**Figure 6:**
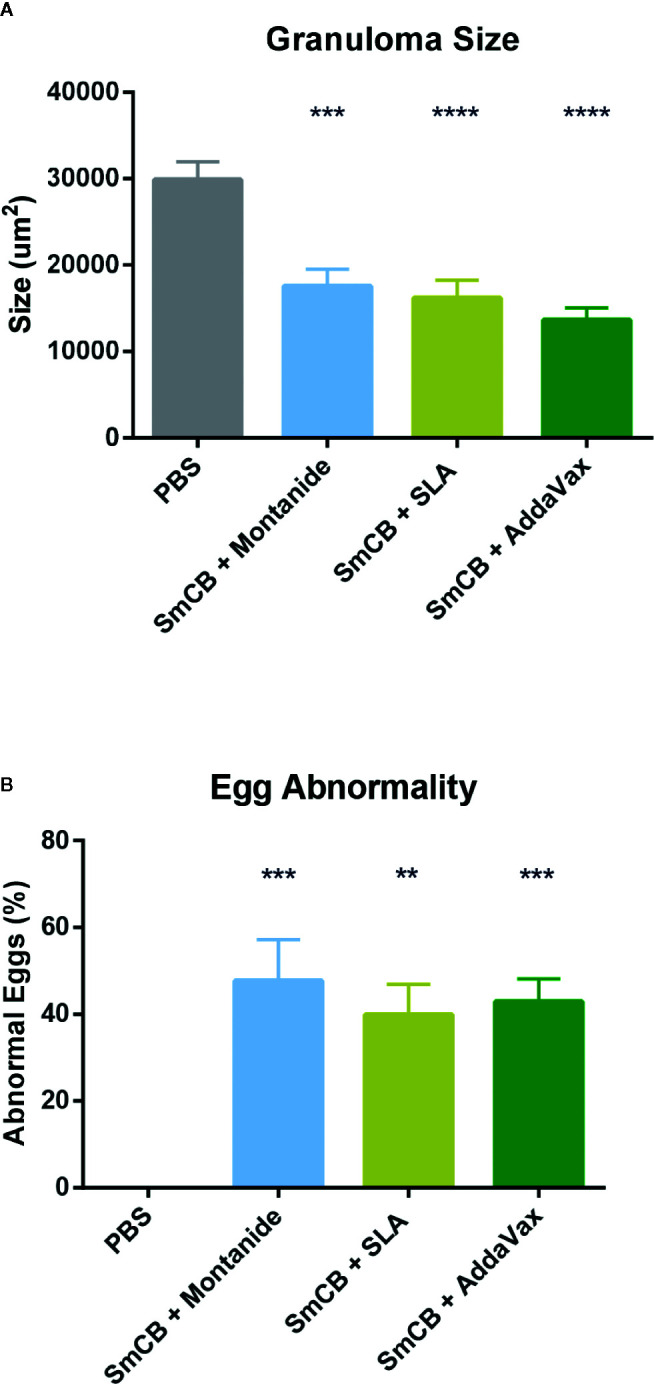
Egg granuloma size and egg abnormality. Using Zen Blue software, 37–41 granulomas were measured per group of vaccinated animals from two independent experiments and the mean and SEM of their size is shown in **(A)**. Of these granulomas, when they were visualized in groups (15 groups of eggs were assessed per experimental group over two independent experiments) a percentage of abnormal eggs was calculated, and the mean and SEM of abnormality is shown in **(B)**. Significance was calculated against the PBS control. **P < 0.01, ***P < 0.001, ****P < 0.0001.

During mouse dissection, images were taken of gross liver sections as pathology was clearly visible (**Figure 5A**). Livers from PBS control mice had many granulomas (visualized as white circular formations) that covered the surface of the liver due to heavy egg deposition, while vaccinated mice in all groups had less granuloma formation compared to PBS controls. By visual examination, mice immunized with rSmCB adjuvanted with Montanide or AddaVax had the least granuloma formation. Microscopic examination of liver tissue stained with hematoxylin and eosin stain (**Figure 5B**) revealed the presence of *S. mansoni* eggs within granulomatous formations. Granulomas were large, and well formed in PBS control mice, and eggs in granulomas were intact with normal appearances. Upon vaccination with adjuvanted rSmCB, granuloma sizes dropped from approximately 30,000 μm^2^ to below 20,000 μm^2^ (**Figure 6A**). Mean granuloma sizes in rSmCB formulated with Montanide and SLA were 17541 ± 1991 μm^2^ and 16185 ± 2070 μm^2^, respectively. Although granulomas were smallest in the group adjuvanted with AddaVax (13637 ± 1398 μm^2^) there were no statistical differences between vaccinated groups. Eggs in vaccinated animals were also abnormal in appearance (i.e., internal structure was lost or compromised, edges were crenellated and incomplete) (**Figure 6B**). A percentage of 47.7 ± 9.5% eggs were found to be abnormal in mice immunized with rSmCB/Montanide. When rSmCB was adjuvanted with SLA and AddaVax, 39.9 ± 7.0% and 42.9 ± 5.3% of eggs were found to be abnormal, again differences between vaccinated groups were not significant.

### Egg Hatching

**Figure 7:**
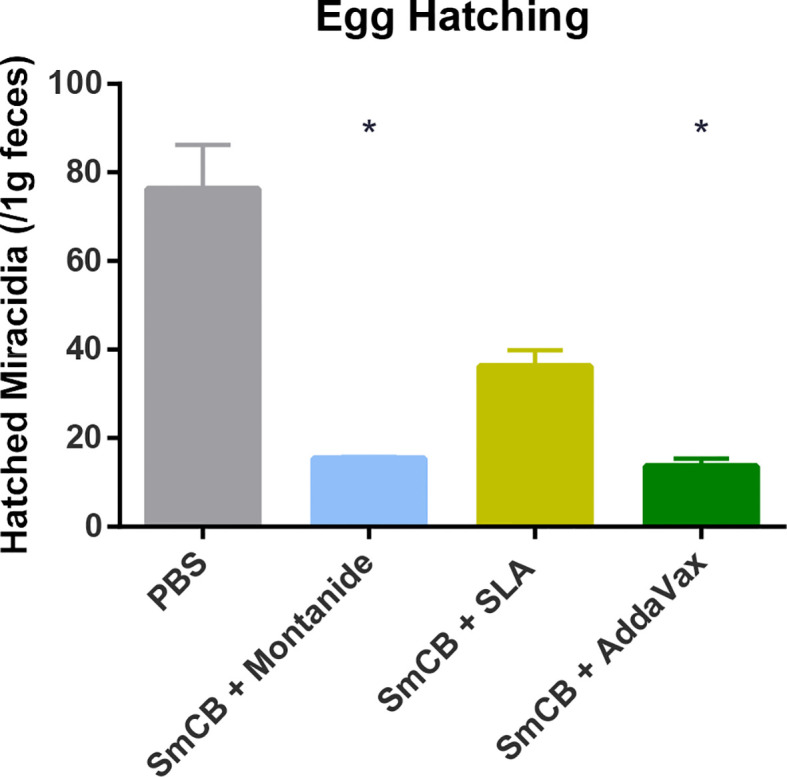
Egg hatching. Seven weeks after challenge, feces from mice were collected and hatched in water. The number of resulting miracidia was counted and adjusted to one gram of feces, and the mean and SEM are shown. Feces were collected from two independent mouse experiments, at two separate time points each. Significance is calculated against the PBS control. *P < 0.05.

To assess whether our vaccine formulations could interrupt the transmission of schistosomiasis we tested whether eggs retrieved from feces were able to give rise to larvae. Feces from PBS control mice gave rise to 76.3 ± 10.0 miracidia (**Figure 7**). Feces from experimental groups saw significant reductions in miracidia: 15.4 ± 0.4, 36.2 ± 3.7, and 13.6 ± 1.7 miracidia hatched from Montanide, SLA, and AddaVax groups, respectively, with no statistical significance between them.

## Discussion

Our group has previously shown the protective capabilities of SmCB, when adjuvanted with CpG dinucleotides (12) and Montanide ISA 720 VG (13). In this work we evaluated the protective capabilities of two new adjuvants: sulfated lactosyl archaeol (SLA) archaeosomes and AddaVax, a squalene-based oil-in-water emulsion similar to MF59. When used as an adjuvant, SLA has been shown to activate strong humoral and cell-mediated responses against multiple antigens by increasing local cytokine production, immune cell trafficking, and antigen uptake at the injection site, leading to increased protection in murine models of infectious disease and cancer (23–25). In this study, we used a novel admixed formulation which provides a simple ready to mix adjuvant formulation with no loss of antigen during the formulation process (26). AddaVax alternatively, is a squalene-oil based emulsion structurally similar to MF59, which acts by stimulating local cytokine and chemokine production, attracting immune cells to the injection site and increasing antigen trafficking and presentation (27).

SmCB, is a gut cysteine peptidase necessary for parasite growth and maturity. Although immunogenic and capable of protecting from *S. mansoni* infection when used alone (28), our laboratory has shown that adjuvants enhance its immunogenicity and protective efficacy (12, 13, 29), the highest protection seen with Montanide (13).

In a series of preliminary studies, we tested a variety of other adjuvant formulations (including: AddaVax, aluminum hydroxide (alum), alum/CpG dinucleotides, alum/monophosphoryl lipid A, and SLA, alongside Montanide and PBS as controls) in combination with rSmCB for immunogenicity and protection from parasite challenge, to determine the most efficacious (**Supplemental Figure 4**). Of the adjuvant formulations tested, the most significant impact on reducing the parasite burden was seen when rSmCB was adjuvanted with SLA or AddaVax. Therefore, the present study was conducted to further elucidate the immune mechanisms behind this protection. The two adjuvanted formulations in this study were able to surpass the WHO schistosomiasis vaccine threshold of 40% protection (6), similar to our previous efforts. SLA reduced adult worms, liver eggs, and intestinal eggs by 60.5, 49.8, and 59.4%, respectively, while AddaVax reached 86.8, 78.0, and 83.4% in the same readouts (**Figure 4**).

Eggs trapped in host tissues release soluble egg antigens triggering granuloma formation, leading to liver cirrhosis and other fatal morbidities (30). Both emulsion-based vaccines (Montanide and AddaVax) were able to visibly reduce granuloma size, and parasite pathology to the liver (**Figure 5**). Granuloma formation is initiated by Th2 immune responses; however, when mice mount extreme Th1 polarization responses, liver pathology is severe (31). This was shown in mice immunized with schistosome egg antigens (SEA) and complete Freunds adjuvant (32), and again in mice that lack both IL-10 and IL-4, which reached 100% mortality upon infection with schistosomiasis (33). Although SmCB is not expressed by eggs trapped in host tissue, it is a secreted protein of *S. mansoni* adult flukes which reside in venules in and around the liver and intestines. It is possible that SmCB specific lymphocyte reactivation is causing the expression of Th1 and inflammatory cytokines that are indirectly contributing to the deleterious liver pathology seen in SLA vaccinated animals. Despite a greater number of eggs found in SLA liver tissues than Montanide and AddaVax, granulomas around these eggs were equally reduced in size.

Eggs released in feces into freshwater, will hatch miracidia, the first larval stage of the parasite. To our knowledge, we are the first group to test *S. mansoni* vaccine efficacy in reducing hatched parasite from fecal samples, although others have demonstrated hatching from liver deposited eggs (34, 35). We found that one gram of feces led to a reduced number of hatched miracidia in animals vaccinated with rSmCB and Montanide or AddaVax. As shown in previous work (9, 36), targeting a digestive enzyme may lead to a suppression of metabolic activities necessary for proper reproduction, leading to the lowered fertility and egg fitness demonstrated by our vaccines. Despite the fact that our results do not account for the variability in fecal egg shedding from day-to-day (37, 38), we believe the reduction in hatched parasite observed in vaccinated animals would mean reduced schistosomiasis transmission.

Immunogenicity studies suggest that the protection mediated by our vaccine formulations could be explained by a robust humoral and cellular mediated immunity (CMI), and it is likely that both these responses contribute to protection from schistosomiasis.

Several groups have shown a positive correlation between IgG antibody titer and protection from schistosomiasis suggesting a necessity for the humoral response. This response was seen to mediate antibody mediated cellular cytotoxicity (ADCC) and activate complement as an attack against schistosomula (29, 39, 40) *in vitro*. By this mechanism or due to another, high IgG titers have been found in vaccinated animals with reduced adult worm burdens. Interestingly, a study in rhesus macaques not only showed a reduction in worm burden correlated to IgG, but collected worms were morphologically stunted with degenerated reproductive systems (41). As our vaccine formulations produced robust IgG titers, they all showed promise for a protective vaccine. Learning from the failed hookworm vaccine (42), we wanted to ensure our vaccine formulations did not cause IgE hypersensitivity, as IgE is a trademark of helminth infections like *S. mansoni* (43). We saw slight increases in total IgE levels after immunization using SLA and AddaVax which were not present in Montanide adjuvanted groups or the PBS control. However, post challenge, total IgE levels were similar in all groups including unvaccinated controls (**Figure 1C**). Thus, detrimental effects associated with vaccine induced IgE responses are unlikely.

Ex vivo re-stimulation of splenocytes with rSmCB showed significant lymphoproliferation in all vaccinated groups, so we were curious to see what cell mediated immunity was being elicited by our different vaccines. Although all vaccine groups increased cytokine expression, there were subtle differences in their cytokine milieus between different adjuvant formulations (**Figure 2B**). When combined with SLA, SmCB was broadly stimulating increasing inflammatory cytokines, Th1 and T-cell associated cytokines, as well as the myeloid proliferation cytokine IL-3, whereas with Montanide and AddaVax, SmCB led to increased Th17, and Th2/Anti-inflammatory cytokines, respectively.

From the creation of the *S. mansoni* radiation-attenuated cercaria vaccine, it has been the consensus that IFNγ and TNFα play pivotal roles in protection (44–46). It is a promising feature that when CD4+ and CD8+ T cells from vaccinated animals were stimulated ex vivo with rSmCB we observed increases of IFNγ, with trends of increased TNFα. Although the percentage increases observed are small, the number of cells that they represent specific to our antigen is significant. Interestingly, our multiplex ELISA data show significant production of IFNγ by all vaccinated groups, which is not fully reflected in our T cell expression as seen by flow cytometry. Future studies could prove useful to identify which other cell types are contributing to IFNγ expression, especially for mice vaccinated with Montanide and SLA.

As previously mentioned, both SLA and AddaVax have been shown to activate the immune system (23–25, 27) and it is due to this quality that they have been exploited as vaccine adjuvants. This study sought to assess the increased efficacy of SmCB when combined with these compounds, however further studies should be conducted to elucidate the possible protective effects of the adjuvants themselves in schistosomiasis infection.

Adjuvanting schistosomiasis vaccines is not a new concept. Previous work has shown 70% reduction in worm burden with a Sm-p80 tegument vaccine administered by DNA prime and boosted with protein and oligodeoxydinucleotides (47), and 57% reduction in worm burden with a Sm-Tsp-2 tetraspanin vaccine adjuvanted with Freund’s incomplete adjuvant (48), among others. However, to our knowledge, we are the only group to test SLA archaeosomes and AddaVax in the presence of SmCB and are reducing adult worm burden the most significantly of all the recombinant protein vaccines in pre-clinical trials. Our data support the hypothesis that *Schistosoma mansoni* Cathepsin B is a strong candidate for an anti-schistosome vaccine and can be readily formulated with multiple different types of adjuvants including oil-in-water and water-in-oil emulsions, archaeosomes and TLR9 agonists. Future directions include conducting dose response experiments on tested adjuvants, as a single dose level of SLA was tested, and AddaVax was formulated as per the manufacturer’s guidelines. Additionally, it would be useful to conduct more in-depth immunological and mechanistic studies to further elucidate the correlates of protection being elicited by our vaccines.

## Data Availability Statement

The raw data supporting the conclusions of this article will be made available by the authors, without undue reservation.

## Ethics Statement

The animal study was reviewed and approved by the Facility Animal Care Committee of the Research Institute of the McGill University Health Center (Animal Use Protocol 7625).

## Author Contributions

Experimental design was conducted by DP and MN in collaboration with YJ, RW, and MM. AR provided the initial protocols needed for protein expression. Experiments were conducted by DP and AH assisted with animal sacrifice. The manuscript was prepared by DP and MN, with revisions by YJ, RW, and MM. All authors contributed to the article and approved the submitted version.

## Funding

This work was supported by grants provided by the Public Health Agency of Canada and the Foundation of the McGill University Health Center.

## Conflict of Interest

The authors declare that the research was conducted in the absence of any commercial or financial relationships that could be construed as a potential conflict of interest.
