# Supplementary material for: Adjuvanted Schistosoma mansoni-Cathepsin B With Sulfated Lactosyl Archaeol Archaeosomes or AddaVax™ Provides Protection in a Pre-Clinical Schistosomiasis Model
Source: Front Immunol. 2020 Nov 16;11:605288. doi: 10.3389/fimmu.2020.605288 (PMC7701121; doi:10.3389/fimmu.2020.605288)
Supplement: Supplementary file 1 [file DataSheet_1.docx]

**SUPPLEMENTAL MATERIAL**


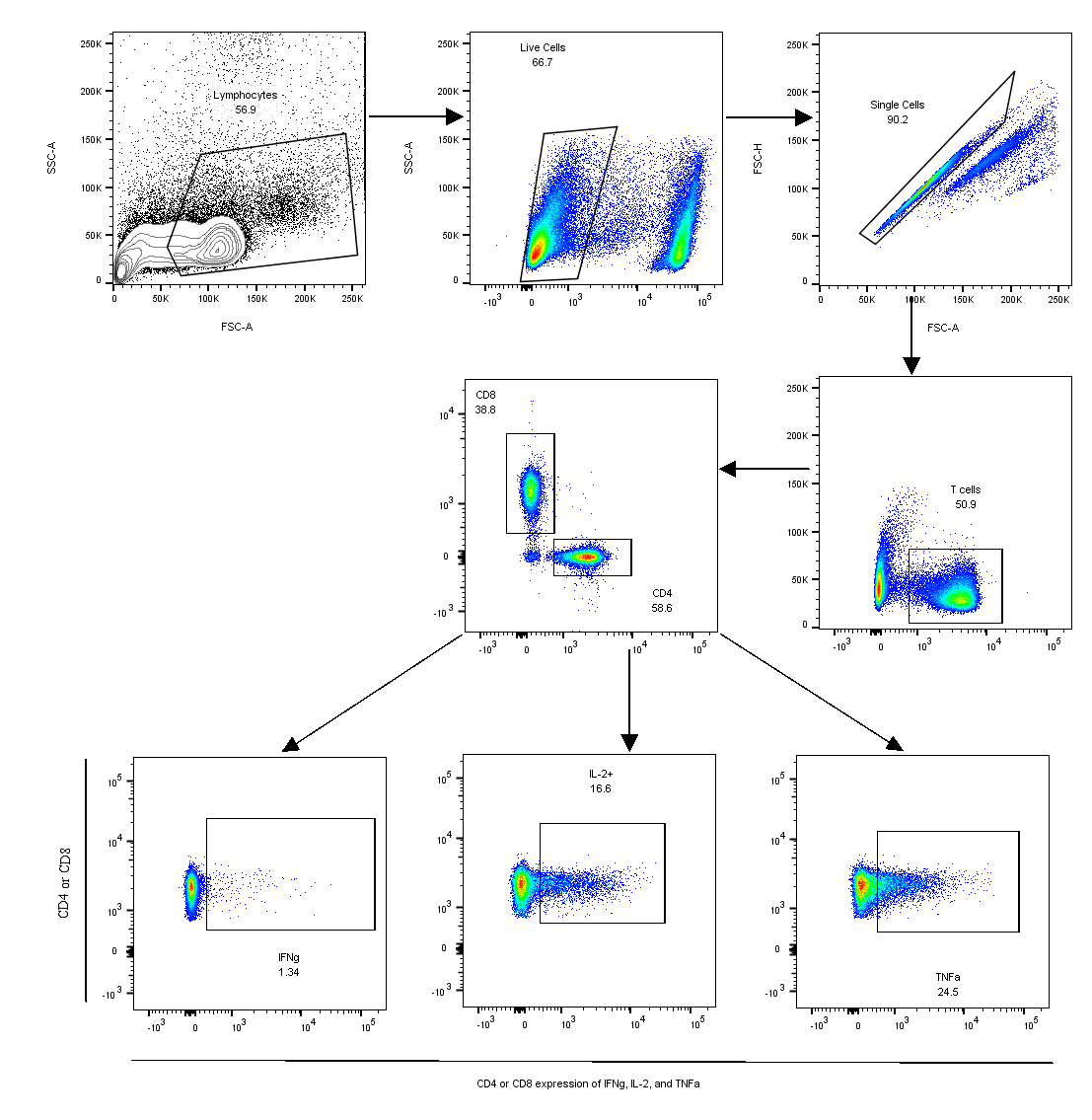


**Supplemental Figure 1.** Gating strategy for flow cytometry analysis. IFNg=IFNγ.

1.
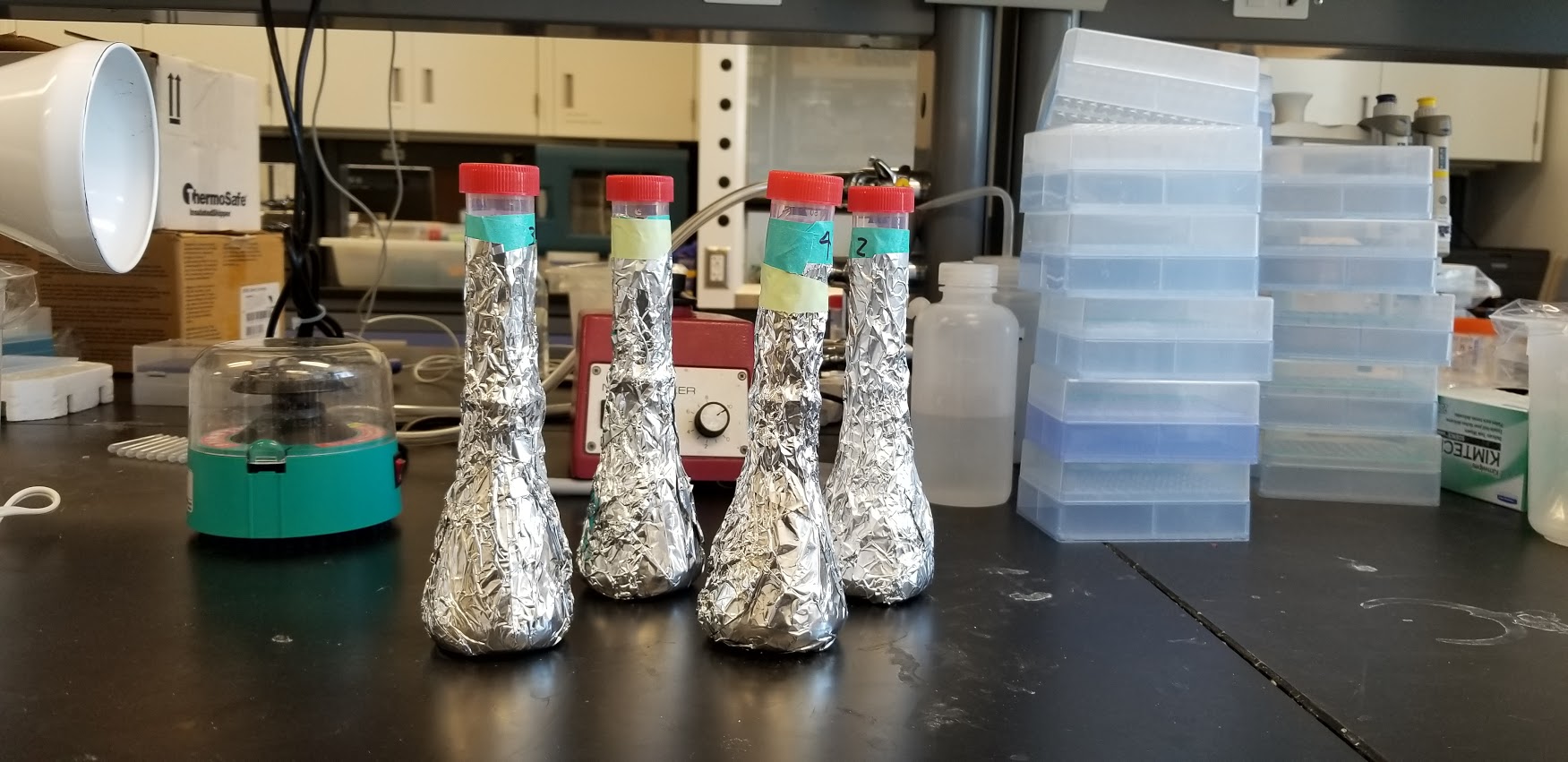

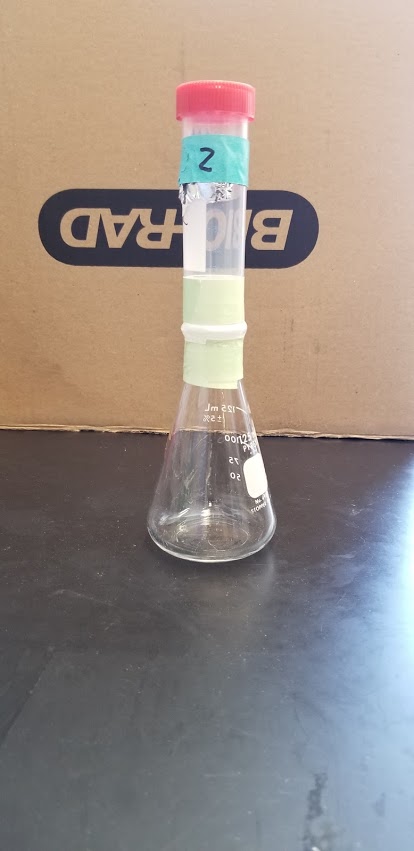
 B)


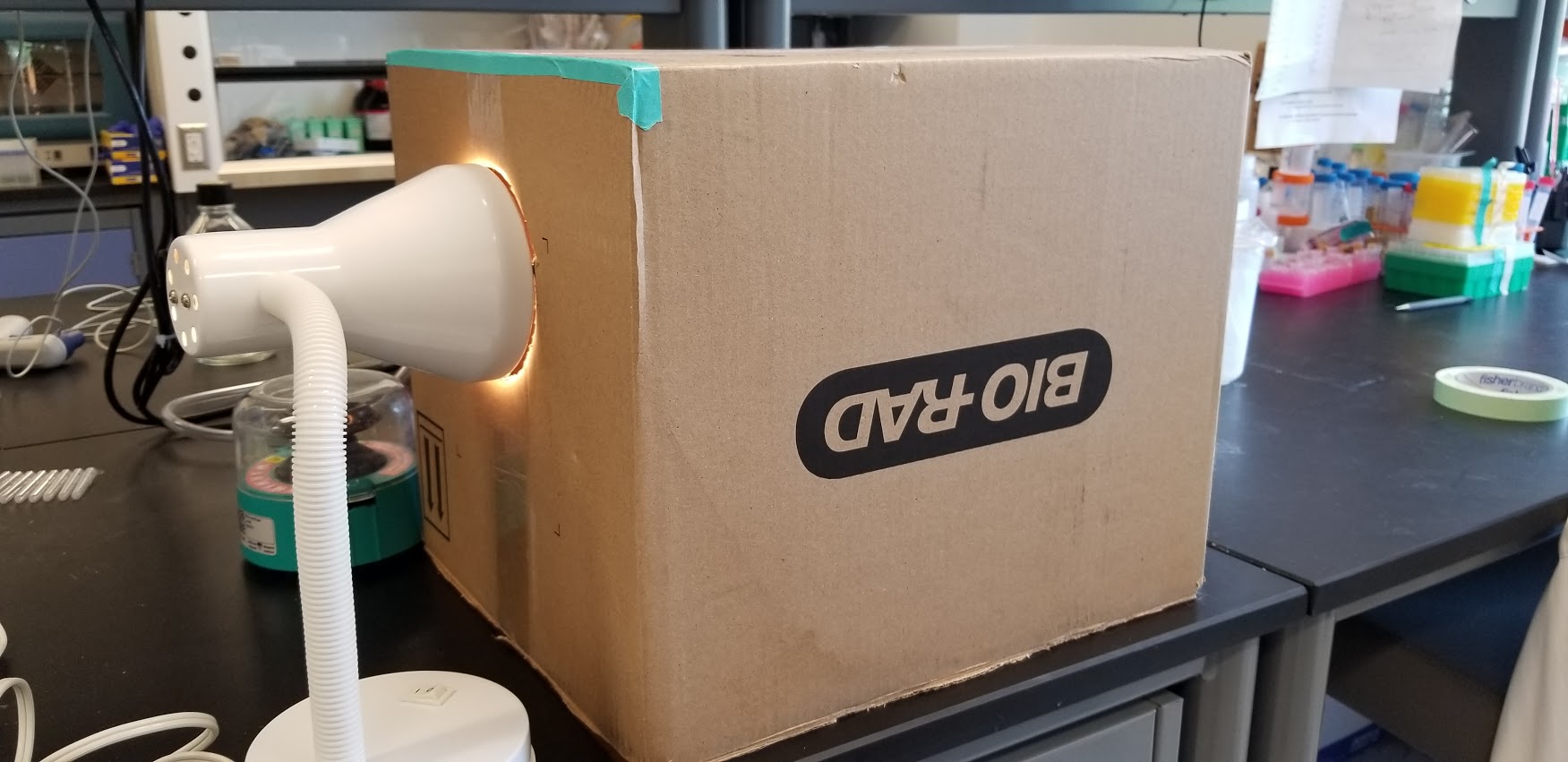
 C)

**Supplemental Figure 2.** Egg hatching set up. Seven weeks post challenge, one gram of feces was put into conical tubes with distilled water. Fecal samples were homogenized then transferred to 125 mL Erlenmeyer flasks. The bottom sections of 50 mL conical tubes were removed using an exacto knife and attached tightly to the top of each Erlenmeyer flask using parafilm and tape **(A)**. The Erlenmeyer flask and tube were covered in tin foil to protect from light, except for the top 3 mm of the tube under the twist-on cap **(B)**. The flask and tube were then filled with distilled water to the top and the cap was attached. These light-protected chambers were put inside of a cardboard box, to further protect from light. A hole was cut into the cardboard box and a lamp was shone into it to direct light at the small sections of unprotected tube (3 mm under the cap) **(C)**. After 3 hours, the top 3 mm of water was removed from the tube using a pipette gun and ejected into a 12-well plate. Hatched miracidia were then counted using a dissecting microscope.

**Supplemental Figure 3.** Cytokine and Chemokine production. N=10 from two independent experiments. Splenocyte supernatants were run on a multiplex-ELISA for 16 cytokines and chemokines: IL-1α, IL-1β, IL-2, IL-3, IL-4, IL-5, IL-6, IL-10, IL-12, IL-17, MCP-1, IFNγ, TNFα, MIP-1α, RANTES, and GM-CSF. Supernatant expression of these molecules can be seen as mean and SEM. Significance is calculated against the PBS control. *P<0.05, **P<0.01, ***P<0.001, ****P<0.0001.

**Supplemental Figure 4.** Pilot study comparing SmCB vaccine formulations. Six to eight-week old female C57BL/6 mice were immunized for parasite burden reduction. Group 1: control: mice were injected with phosphate-buffered saline (PBS) (Wisent Bioproducts, St. Bruno, QC) n=15. Group 2: positive control: mice were immunized with 20 μg of recombinant SmCB (rSmCB) and 35 μL of Montanide ISA 720 VG (SEPPIC Inc., Fairfield, NJ) n=15. Group 3: mice were immunized with 20 μg rSm-CB n=5. Group 4: mice were immunized with 20 μg rSmCB and 25 μL of AddaVax™ (InvivoGen, San Diego, CA) n=5. Group 5: mice were immunized with 20 μg rSmCB and 40 μg of aluminum hydroxide (alum; Alhydrogel; Brenntag BioSector A/S, Frederikssund, Denmark) n=5. Group 6: mice were immunized with 20 μg rSm-CB and 40 μg of aluminum hydroxide and 10 μg CpG dinucleotides (Hycult Biotechnology B.V., Netherlands) n=5. Group 7: mice were immunized with 20 μg rSmCB admixed with 1 mg of pre-formed empty SLA archaeosomes (NRC, Ottawa, Canada) n=5. Group 8: mice were immunized with 20 μg rSmCB and 40 μg of aluminum hydroxide and 10 μg monophosphoryl lipid A (List Biological Laboratories, California) n=5. Each mouse was immunized at weeks 0, 3, and 6 intramuscularly in the thigh with 50 μL of vaccine. Mice were infected at week 9, and at week 16 parasite burden was assessed. Data are shown as mean and SEM. *P<0.05, **P<0.01, ***P<0.001, ****P<0.0001.
